# “Punched in the Balls”: Male Intimate Partner Violence Disclosures and Replies on Reddit

**DOI:** 10.1177/15579883211039666

**Published:** 2021-08-20

**Authors:** Marudan Sivagurunathan, David M. Walton, Tara Packham, Richard Booth, Joy MacDermid

**Affiliations:** 1 Department of Health and Rehabilitation Sciences, Western University, London, ON, Canada; 2 Department of Physical Therapy, Western University, London, ON, Canada; 3 The School of Rehabilitation Sciences, McMaster University, Hamilton, ON, Canada; 4 Health Information Sciences, Western University, London, ON, Canada; 5 Hand and Upper Limb Centre Clinical Research Laboratory, St. Joseph’s Health Centre, London, ON, Canada

**Keywords:** males, intimate partner violence, disclosure, Reddit, social networking sites

## Abstract

Research on male intimate partner violence (IPV) survivors is limited. The sparse research on male IPV disclosure suggest males receive more negative and less helpful responses from potential sources of formal or informal support. Males may seek support on social networking sites (SNSs), hence, it is important to understand their emerging experiences of virtual disclosures. This study examined the nature and content of responses to IPV disclosures by male IPV survivors on a popular SNS (reddit.com). Search of Reddit submissions related to male IPV were carried out using three IPV related keywords for the calendar month of February 2019, resulting in 917 submissions. Twelve submissions that focused on male IPV disclosure were examined in detail. The 12 submissions were analyzed using quantitative content analysis while associated comments (*n* = 569) were analyzed using qualitative approach. Two-thirds of the disclosures (8/12) were of personal IPV experiences. All disclosure narratives identified the sex of perpetrator, most stated the types of abuse (7/12), and some revealed the outcomes of past disclosures (4/12). Six major themes were developed through qualitative analysis of the associated comments: (1) Informational Support, (2) Nurturant Support, (3) Tangible Aid, (4) Negative Response (5) Self-Defence, and (6) Reciprocal Disclosure. Overall, males experienced a majority of supportive responses to IPV disclosures and some negative responses including criticism and minimizing the abuse. Males take risks in disclosure of IPV in person and online.

Intimate partner violence (IPV) can negatively impact an individual’s personal security, identity, agency, and interpersonal connectedness. IPV has been associated with significant economic costs resulting from medical care, lost productivity of victims and perpetrators, as well as legal costs (Peterson et al., 2018).

While IPV is known to be most prevalent in females, males can also experience IPV victimization. Studies indicate health outcomes (e.g., depression, post-traumatic stress disorder, and binge drinking) for male IPV survivors can be devastating (Cerulli et al., 2014; Hines & Douglas, 2015, 2016). A growing body of work suggests IPV prevalence rates are higher in males than originally presumed (Hines & Douglas, 2016; Kamimura et al., 2014). Hines and Douglas (2016) reported that surveys on partner violence show males are the victims of 25%–50% of all physical partner violence within any given year.

Studies of men’s help-seeking behavior highlight the reticence to seek help for diverse issues including, substance abuse, depression, and even milder issues such as concerns about school work or issues with parents (Farrand et al., 2007; Yousaf et al., 2015). Conformity to traditional masculinity, shame, and embarrassment are consistently cited as barriers to help-seeking amongst males, including male survivors of IPV (Machado et al., 2016; Walker et al., 2020). A systematic review by Tsang (2015) attributed male IPV survivors’ reluctance to seek help to both; (a) internal barriers including attempts to maintain their masculinity, shame, a belief in taking responsibility for the children, internalized societal expectations regarding gender roles, fear of not being believed, and perceptions of limited resources, and (b) external barrier including limited services available for male IPV survivors. Studies on social reaction to disclosures of IPV victimization by survivors are limited. Within this limited area of research, findings suggest that female survivors of IPV are more likely to receive positive reactions to disclosures compared to males (Edwards & Dardis, 2020). Some male IPV survivors reported unhelpful responses from social and victim services, police, and the justice system (Machado et al., 2016, 2020; McCarrick et al., 2016; Walker et al., 2020).

Help-seeking in an online community may remove some of the barriers and stigma associated with face-to-face help-seeking. Factors associated with online communication can serve to reduce inhibition and make it more likely for individuals to disclose sensitive information (Suler, 2004). Users who feel stigmatized find the anonymity presented by online support communities to be helpful (Barak et al, 2008) and allow users to ask questions or express themselves without shame (Tanis, 2008).

As internet availability and usage increases, there has been a greater global trend towards sharing one’s personal life online. Social networking sites (SNSs) offering some degree of perceived anonymity can provide male IPV survivors a platform to disclose their IPV experiences and seek support without the risk associated with other more public disclosure approaches. Reddit is a suitable platform for exploring the topic of male IPV due to multiple factors. Reddit is one of the largest SNSs with over 430 million average active monthly users, and 21 billion average screen views per month^1^. Users from more than 200 different countries access reddit.com (Sharma et al., 2017). Reddit is not constrained by a small character or word limit, boasting a generous 40,000-character limit which is important for rich data in qualitative studies. Unlike some of the other SNSs which encourage users to create accounts with real names, Reddit allows for both pseudonym accounts and also “throwaway” accounts—temporary accounts created for a specific purpose—and are “used as proxies of anonymity” (Pandrekar et al., 2018, p. 867). Subsequently, the anonymous nature of the throwaway accounts may allow users to discuss sensitive topics and be more candid in their discussion (Pandrekar et al., 2018).

Given the benefits and harm associated respectively with positive and negative responses to disclosures of IPV, coupled with the increasing use and availability of SNSs and desire of male IPV survivors to disclose their status in anonymous fashion online, there is an opportunity to examine how male IPV survivors discuss their experiences via social media. Disclosure and responses of male IPV on SNSs such as Reddit may differ from face-to-face or other traditional disclosure interactions. The findings of the current study are part of a larger project which sought to explore the discourse around male IPV on Reddit. The purpose of the current study is to examine the disclosures and the related replies to self-disclosure of male IPV as it happens on Reddit.

## Methods and Study Design

### Data Collection

Python Reddit Application Programming Interface (API) Wrapper (PRAW) which allowed access to Reddit’s API was used in order to collect, or “scrape,” the data from Reddit. In February 2019 the PRAW was used to carry out a search of all Reddit submission titles and submission bodies using three separate keywords (“male IPV,” “male domestic abuse,” and “male domestic violence”). The search provided the authors with the unique identifying code (submission ID) associated with each submission that contained the keywords in either the submission title or submission body. The submission IDs were then used to collect the submission, submission engagement metrics (i.e., upvote, upvote ratio, number of comments), and all associated comments. As we were using publicly available data, the study did not meet the requirements for formal institutional ethics approval through university review board.

### Submission Data

**Figure 1.**
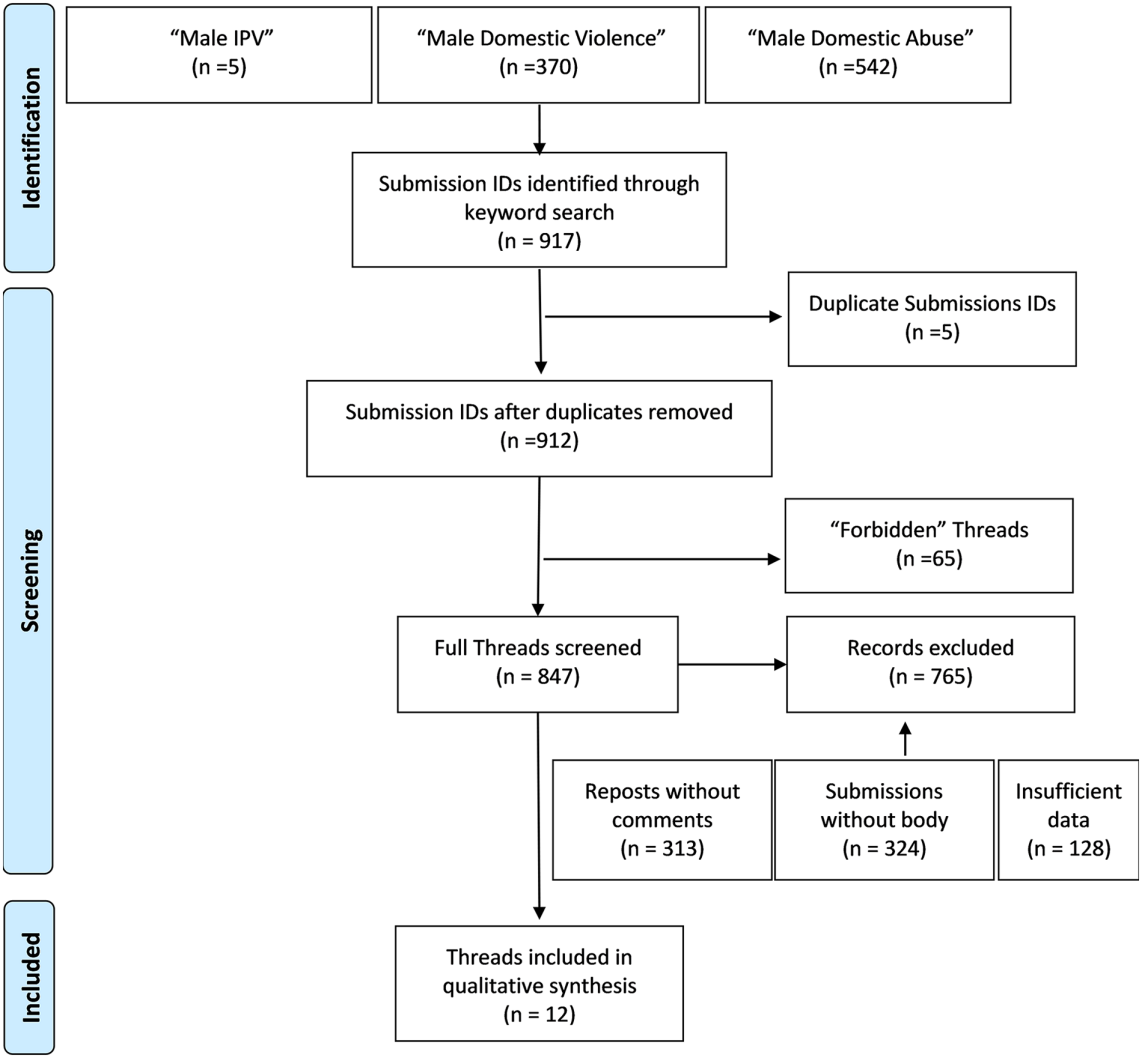
PRISMA figure of submission inclusion criteria.

The search produced 917 submission IDs which included the search ketwords in either the submission title or body (see Figure 1). Five duplicate submission IDs were removed while 65 submission IDs were excluded as access to complete thread was “forbidden” by Reddit due to site privacy restrictions. Submissions titles, submission body, and associated comments for all 847 threads were collected and screened. Through screening some of the threads were removed for being reposts with no associated comments. Some threads were removed for not having a submission body-this included cases where submission body was removed by the administrators of the subReddit, submission body was deleted by the author of the submission, or submission body consisted of external links to YouTube, newspaper articles, research articles, or other sources. Some threads were excluded for having insufficient data, which was operationalized as a thread with less than half a page of data pertaining to male IPV as these short threads lacked sufficient contextual details or description to fully appreciate/interpret the nature of the discussion. This resulted in a final set of 82 threads that were assessed for eligibility. To be eligible the submission had to contain a disclosure of male IPV (personal experience or experience of friend/family/other). The final set of data contained 12 submissions (see Table 1), and associated comments (*n* = 569).

**Table 1.** Summary of Submissions Disclosing Male IPV.

IPV disclosure	Number of comments	Upvotes	Upvote ratio	Brief summary of presenting submission
Submissions with disclosure of personal IPV experience				
Submission #1	9	6	0.88	Account of gay man’s disclosure of IPV and its impact on him and his perpetrator.
Submission #2	139	643	0.90	Account of IPV by wife, false allegation, worry about child, divorce.
Submission #3	6	4	0.75	Account of disclosing his IPV by female partner on a radio talk show and its consequences.
Submission #4	3	6	0.72	False allegation by female partner. Looking for financial help to settle legal case.
Submission #5	7	11	0.74	Account of biased IPV campaign silencing him from disclosing his own IPV by female partner.
Submission #6	70	196	0.97	Looking for help (financial, moving) to leave abusive female partner.
Submission #7	272	105	0.80	Looking for other males with experience of IPV by female partner.
Submission #8	31	61	0.85	Looking for other males with experience of IPV by wife.
Submissions with disclosure of IPV experience of male family member/friend/other				
Submission #9	9	6	1.0	Gay man seeking advice on how to help ex-boyfriend deal with abuse by ex-boyfriend’s current partner.
Submission #10	10	23	0.87	Medical student providing care to male patient admitted to hospital due to IPV injury by wife.
Submission #11	4	33	0.88	Account of IPV experience of an acquaintance remaining in an abusive marriage due to children.
Submission #12	9	0	0.13	Seeking clarification and advice on friend’s IPV by friend’s female fiancée.

### Data Analysis

The final set of data was imported in to NVivo (QSR International Pty Ltd. Version 12, 2018) for data analysis. The data analysis was carried out in two phases: (a) quantitative content analysis of the initial disclosure of male IPV by original poster (OP); and, (b) thematic analysis of the comments associated with the submissions. During both phases first author (M.S.) carried out the coding with all the authors meeting throughout the process to discuss the codes and themes identified by first author and any assumptions or biases.

#### Quantitative Content Analysis of Submission

Descriptive data (number of comments, upvotes and upvote ratio) were obtained through scraping Reddit. We utilized quantitative content analysis to code the disclosures by OP. A codebook was developed a priori and was informed by previous research on male IPV survivors. Codes related to “Seeking Support” were derived from the Social Support Behaviour Code (SSBC) developed by Cutrona and Suhr (1992) “to assess social support behaviors in the context of help-intended dyadic interactions in which one member of the couple discloses a personal problem to the other”(Kerig & Baucom, 2004, p. 307).

#### Thematic Analysis of Comments

Phase two consisted of analyzing the comments associated with the disclosure submissions using a form of thematic analysis (template analysis) as outlined by Brooks, McCluskey, Turley, and King, (2015). This thematic approach permitted inductive analysis to expand on the deductive approach of the content analysis in Phase One. The initial coding process consisted of creating an a priori codebook of themes and codes informed by an existing framework on social support, the SSBC (Cutrona & Suhr, 1992). Through the coding process new codes that did not fit into the themes present in the initial codebook became salient and these emergent codes were added to the codebook.

## Results

### Characteristics of IPV disclosures by male users of Reddit

Quantitative content analysis of the submissions (*n* = 12) reflect the complex nature of IPV disclosure as multiple content categories emerged across each disclosure. See Table 2 for complete list of content categories.

**Table 2.** Quantitative Content Analysis of Disclosure Submissions (n = 12).

**Content Category**	**No. of Submissions**
**Facets of IPV experience**	
Type of disclosure	12
Disclosure of personal abuse	8
Disclosure of abuse experienced by others	4
Gender of perpetrator	12
Female	10
Male	2
Emotions	6
Fear	4
Shame	3
Sadness	1
Loneliness	1
Hope	1
Types of abuse	7
Physical	7
Emotional	4
Isolation	3
Control	3
Legal/administrative	3
Verbal	2
Financial	1
Contextual factors	3
Alcohol	2
Politics	1
Impact of abuse	3
Suicide attempt	2
Stress	1
Financial issues	1
**Reasons for disclosure**	
Seeking support	8
Informational support	4
Social network support	3
Tangible aid	2
Raising awareness	1
**Dimensions of IPV disclosure**	
Outcomes of past disclosure	4
Negative response	4
Positive response	2
Abuse narrative	3
Self-blame	3
Identifying victimization	2
False allegation	3
Systemic bias	4

*Note:* More than 1 content category can appear in each submission.

#### Facets of IPV Experience

Most of the submissions on disclosure (*n* = 8) consisted of disclosing personal experience of IPV, while the rest of the disclosures (*n* = 4) included IPV experience of friends (*n* = 2), ex-boyfriend (*n* = 1), and a patient (*n* = 1). Females were identified as being the perpetrator in majority of the submissions (*n* = 10).

Some of the submissions (*n* = 6) explicitly referenced emotions associated with the IPV experience or disclosure. These emotions identified by the submissions included four negative emotions (i.e. sadness, loneliness, shame/embarrassment, and fear) and one positive emotion (i.e hope). Men expressed fear of: the abuser, the abuser finding out about the disclosure, false allegations, not being believed by friends and family, and losing their children. Men also expressed they were hopeful the abusive situation was temporary and that they would come of out if it stronger (*n* = 2).

Some of the disclosure submissions (*n* = 7) contained the description of the type of abuse they had experienced. Physical abuse was the most prevalent type of abuse disclosed by the men (*n* = 7). Some men (*n* = 3) disclosed experiencing controlling behaviors which included perpetrators not allowing the men to socialize with friends and family, checking their phone and social media accounts, taking away their phones and other means of communication, and monitoring men’s time.

While most (*n* = 9) submissions did not provide a context for abuse, alcohol (*n* = 2) and politics (*n* = 1) were identified as the trigger for the abuse by the men who did identify a reason. Impact of the IPV victimization included survivors attempting suicide (*n* = 2), facing financial burden (i.e., becoming homeless, losing their job; *n* = 1), and stress (*n* = 1).

#### Reasons for IPV Disclosure

While one submission indicated that they were disclosing their abuse to raise awareness of the issue of male IPV in majority of submissions(n=8) men were seeking some form of support. Three of the support seeking themes (i.e., informational support, social network support, and tangible aid) described by Cutrona and Suhr (1992) were identified. Informational support seeking (*n* = 4) consisted of men looking for advice on how to deal with their personal IPV situation or how to help someone else who is dealing with male IPV. Informational support took the form of indirect question such as, “I feel like there’s something else I should be doing, but I have no idea what. . . It’s infuriating” or direct questions, such as, “I’m just wanting some help or advice.” Social network support (*n* = 3) consisted of submissions where the men sought out other male IPV survivors to relate with or share their experience with. For example, one poster indicated, “My wife has been violent towards me and I’d love to talk to someone anonymous about it. Willing to humor me? PM me or just reply to this post.” Finally, tangible aid (*n* = 2) consisted of men seeking financial aid so they can leave the abusive relationship as well as help with moving out.

#### Dimensions of IPV Disclosure

Submissions that mentioned having previously disclosed their IPV experience (*n* = 4) indicated having received both positive and negative responses. Disclosure had been made to friends and family (*n* = 4), police and court personnel (*n* = 2), IPV services (*n* = 2), and healthcare professionals (*n* = 1). Men (*n* = 4) reported receiving negative response from friends and family, police and court personnel, and health care professionals. Negative responses from friends and family included lack of belief, blaming the victim, and anger directed at the victim for disclosing IPV expereince. However, participants also mentioned positive responses from friends (*n* = 1) and IPV services (*n* = 2).

Identifying victimization and self-blame emerged as part of abuse narrative across some submissions (*n* = 3). Some men (*n* = 2) had trouble identifying the victimization or accepting the relationship as abusive, with one man posting, “Punched in the balls, still not sure if I’m in an abusive relationship.” Men (*n* = 3) also blamed themselves for being in the abusive relationship or triggering the abusive incident. These men attributed the abuse to their personality, “I might have personality traits that predispose me to being taken advantage of. I need to work on that. Maybe I’m eager to please, easily swayed, liking forward women etc,” or to their own behavior, such as, “I was perhaps dismissive of my wife’s emotions during her lows.”

False allegations were also a dimension of IPV that emerged across some submissions (*n* = 3). Experiences included instances where a female perpetrator made the false allegation to either the men or to the police. One submission, speaking about their experience of false allegation, indicated, “I am one of the many stories of domestic abuse by their female partners and one who was actually falsely accused and now in dire threat to serve 6 to 20 years in jail for something I did not do.”

Finally, some of the men (*n* = 3) recounted experiences of systemic biases. Given the breadth and depth of this dimension of disclosure it has been explored in detail elsewhere (Sivagurunathan et al., 2021).

### Spectrum of Support by Reddit Users

**Figure 2.**
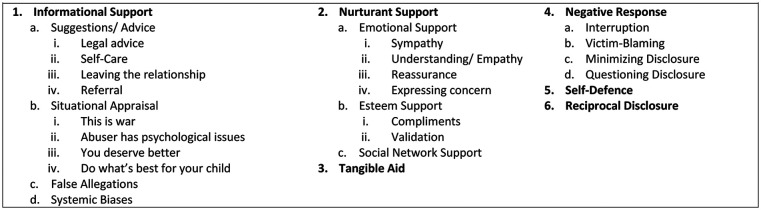
Final version of the template.

Six major themes were developed through thematic analysis of the comments (*n* = 569) associated with the 12 submissions; (1) Informational Support, (2) Nurturant Support, (3) Tangible Aid, (4) Negative Response (5) Self-Defence, and (6) Reciprocal Disclosure (see Figure 2).

#### Informational Support

Informational support can be characterized as “behavior that provides information to the person under stress about the stress itself, about how to deal with the stress, or about how to appraise the situation” (Jensen, 2001, p. 109). The theme consisted of 4 major subthemes: (a) Suggestion/Advice; (b) Situation Appraisal (c) False Allegation, and (d) Systemic Biases

##### Suggestion/Advice

This subtheme represents how commenters provided a course of action to deal with the IPV. Suggestion/advice took either a direct form or was indirectly supplied through a story. Indirect forms of suggestions/advice typically consisted of disclosure of personal IPV experience or IPV experience of friends/family and how the IPV survivor dealt with the abuse. For example, one commenter whose advice was to document the OP’s interaction with the abuser noted:

I have seen this entire situation play out with my husband’s best friend. He was arrested for domestic violence, even though he never touched her. Now he has a record and can’t get the job he went to school for. Sad thing is, he is one of the nicest people I know. I would record every interaction you have with her. EVERY single interaction.

Advice provided by commenters fell in a broad spectrum and included: legal advice, self-care, leaving the relationship, and referrals.

In terms of legal advice most of the commenters expressed that OP should minimize or avoid contact with the abuser entirely. If contact with the abuser could not be avoided, then the commenters advised documenting the abuse or any contact with the abuser as well as having a witness present when meeting with the abuser. This advice stemmed from commenters’ personal experience of being falsely accused or commenters fearing that the OP might be falsely accused, as illustrated in this response:

buy a voice-activated recorder and carry it at all times. You may not be able to use it in court but I know a few men who have had their hides saved by the evidence. Even better, nannycams, webcams, and cell phones on record are also excellent ways to demonstrate that you’re not the one who hit first.

In addition to preventing false allegations commenters suggested that documenting the interactions and having witnesses could be beneficial in family court. Commenters advised seeking full custody in order to ensure children’s safety and healthy development, suggesting that documenting the interactions could prevent abuser from making false claims in family court, as one commenter advised:

Text her that you are leaving the house for 30 minutes. That way she cannot claim in the future that she tried coming to the house with your daughter for a visit and you weren’t there. Save these messages as proof that you are trying to communicate and she isn’t.

Another commenter suggested documenting the abuse given the biases they perceived as present in the justice system, indicating, “Make sure you record her being abusive. The police still aren’t that open minded about male domestic abuse.” Nevertheless, despite perceptions of systemic biases, commenters still advised OP to seek help from the police and legal system. Commenters advised that police officers can “escort you to the residence, and watch over you while you pack up your own stuff without being hassled.” Commenters also suggested that police officers can help provide referrals to IPV services, and help establish men’s IPV experience “even if it’s just a case of getting some contact on record.” Commenters also stressed the importance of OP having good legal council to protect themselves from false allegations. One commenter remarked:

I’d be inclined to call a lawyer first and discuss your options before I called the cops, since there’s been some nasty precedents set in situations like this, specifically where a man gets abused, calls the cops, and gets arrested himself even if he’s the only one with injuries.

Responses also urged OP to be as honest as possible and to not “hold back or gloss things over” and that OP should “stop trying to be Mr. Nice Guy.” Commenters felt that given the possibility that the female aggressor would make false allegations, glossing things over to protect the abuser would work against the OP when interacting with the police and judicial system and felt downplaying the abuse would also be problematic in custody hearings.

Commenters also provided OP with self-care advice including seeking support from one’s social circle, seeking help from healthcare professionals, and healthy lifestyle habits. They stressed the importance of reaching out to friends and family for support during episodes of abuse or during separation. Other suggestions included that OP should “hang out with friends and family” and not “be afraid to lean on family and close non-mutual friends” to reduce stress. Commenters also suggested survivors of abuse “should see a therapist it isn’t healthy to bottle stuff up even if you are dealing with it correctly.” The commenters suggested that mental health professionals would be helpful in not only dealing with the emotional trauma of the abuse but also provide support during separation. In addition, commenters suggested speaking to physicians to receive medications to help alleviate any physical or emotional issues.

Finally, commenters stressed the importance of proper diet and rest as well as maintaining healthy lifestyle habits, with one commenter replying, “Keep eating, sleeping, and drinking water dude. Seriously, it’s easy to forget while dealing with persistent anxiety, and you might not recognize the symptoms of malnutrition, dehydration and sleep deprivation once they’ve already manifested.” Other commenters expressed the benefits of exercise on mental health and reducing stress and anxiety and suggested that OP “Get some exercise too, it helps burn off the anxiety.”

Commenters also advised the OP to leave the relationship as soon as possible. They expressed that it would be better to leave the abusive relationship rather than staying and trying to reform the abusive partner. Some commenters indicated that the OP should, “man up and leave the abuser.” Others presented a more supportive perspective that OP deserved to find someone who truly loved them and staying in the abusive relationship would prevent OP from finding someone to have a healthy relationship with. When OP mentioned having a child, commenters expressing concern for the child urged them to leave the relationship for the sake of the child.

Finally, despite highlighting some perceived systemic issues around services for male IPV survivors, commenters advised OP to seek support from services for their IPV victimization. Commenters provided general advice that OP reach out to IPV support services and educate themselves about male IPV victimization. Commenters advised OP that services can provide tangible help, such as “help you escape, either by referring you to a refuge or hostel, or helping you to find a privately rented flat,” or provide informational support, suggesting, “you can get in touch with one of the agencies that specialize in this kinda thing, they’ll be able to give you something more accurate of what will happen.” Commenters also provided referrals to specific organizations/services, as well as online communities/blogs/and websites that provide information and education as well as providing support for male IPV survivors. For example, one commenter expressed:

There are a lot of associations that can help him get out if he’s ready. I don’t know what or where they are in Texas (a quick google search led me [to this site] (http://www.tcfv.org/resources/resources-for-survivors/) and [this hotline] (http://www.loveisrespect.org) and [this list] (http://www.ncdsv.org/ncd_linkstexas.html) and also [this one] (https://www.hhsc.state.tx.us/Help/family-violence/centers.shtml).

##### Situational Appraisal

Subtheme was developed from comments providing a perspective on the victimization. These perspectives included the belief that this is war, abuser has psychological issues, OP deserves better, and OP should do what’s best for their child.

Commenters urged that OP should perceive the situation as a war. They stressed the perpetrator would try to convince friends, family, society, and the legal system that they were the victim and advised the OP; “put on your battle armor and consider this war. She already does.” When OP disclosed having a child with the perpetrator commenters likened child custody hearing to a battle, “it’s pretty much a legal (uphill) battle, so you can drop the gloves and wage all-out war.” Commenters warned against being nice or underplaying the abuse and stressed that it is important “to get more cutthroat.”

The belief that the perpetrators’ abusive behavior stemmed from some form of psychological issue was common. Commenters advised the OP to, “find out of she has a history of mental illness” and to talk to the psychiatrist about diagnosing and medicating the perpetrator. Commenters also provided possible diagnoses as causes of the abuse (e.g., Bipolar Personality Disorder, Histrionic Personality Disorder, and Narcissistic Personality Disorder). For example, one commenter posited, “From her emotional outbursts, it sounds like she could have bipolar disorder or some other sort of mood disorder,” while another indicated, “your wife sounds like she has narcissistic personality disorder.” Commenters provided links to mental health resources and urged OP to educate themselves on how to deal with such individuals.

Sentiment that the male survivors of IPV deserves to find happiness and do not deserve the abuse or to put up with an abusive partner were expressed by many of the commenters. Commenters remarked that male IPV survivors “are not worthless and do deserve help.” Responses also stressed that the OP deserves to find true love and that staying with the perpetrator will prevent OP from finding someone who truly loves them and is supportive. One commenter remarked, “if you want to ever meet someone who will love you then you must move on from her.” Commenters expressed that the OP should not confuse abusive behavior with love, suggesting that if the perpetrator truly loved OP they would not abuse towards OP. One commenter observed:

Here’s the thing. If your partner belittles/demans (sic) you, strikes you, raises their voice at you in anger or frustration, you probably aren’t with the right person. people who love each other shouldn’t feel the need to do those things.

In cases where the OP indicated that they had a child with the abusive partner, the commenters urged OP to act for the benefit of the child. Once commenter indicated, “It’s obvious she doesn’t have the best interest of the child as a priority.” Another response highlighted the potential effects of witnessing IPV:

Keep your daughter in mind when you waver, too. She shouldn’t grow up seeing this. She shouldn’t grow up learning that this is what love is, that this is how a person treats another. Being exposed to abuse is damaging, even if she hasn’t been hit (yet).

Others believed that the abuser may eventually turn on the child, commenting, “Your daughter will be the one that is being chased with a knife/choked/hit.” Commenters stressed that it was important for OP to leave the relationship to protect the child from being a victim of violence.

##### False Allegations

The notion of false allegations emerged across both suggestions/advice and situational appraisal subthemes. While false allegations were raised as warnings in the context of suggestions/advice, situational appraisal comments focused on the potential risk of false allegations by females leading to male IPV survivors being disadvantaged in multiple ways. Commenters noted that females may play the victim and falsely accuse the OP of perpetrating the IPV in order to gain sympathy from law enforcement, court system, and social service organizations. Commenters alleged females would falsely accuse males in order to have an advantage in custody and/or divorce cases, or to avoid being arrested or sentenced for their IPV.

##### Systemic Biases

Commenters with their own experiences of IPV indicated experiencing and being further victimized by systemic biases, including being arrested as a perpetrator, losing custody of their children, and being turned away from services. Commenters provided suggestions/advice to help navigate the social, healthcare, and legal spheres without being further marginalized due to existing systemic biases. Furthermore, OP was advised to take systemic biases into account when considering their situation and actions. Some commenters identified systemic biases as a major barrier to disclosure of male IPV. These perceptions of systemic biases have been explored in depth elsewhere (Sivagurunathan et al., 2021).

#### Nurturant Support

Nurturant support (Baucom & Kerig, 2004) is composed of three subthemes that elicit warmth, and consist of comments having a positive emotional tone: (a) Emotional Support, (b) Esteem Support, and (c) Social Network Support.

##### Emotional Support

The subtheme of emotional support was developed to synthesize the solicitous elements of sympathy, understanding/empathy, reassurance, and expressing concern. Sympathy for the abuse experienced by OP was expressed through comments such as, “Sorry for your situation” or, “It really sucks you have to go through this.” The commenters commiserated with the OP’s experience of IPV, having to be separated from their children, and for the negative emotions such as loneliness or sadness that the OP may be experiencing due to the violence or separation from the abusive partner.

Responses also expressed understanding/empathy regarding the IPV and the resultant fallout. Commenters voiced empathy with replies such as, “I feel your pain” or, “I know it’s hard.” In some cases, commenters used reciprocal disclosure to situate their understanding of OP’s IPV experience. Reassurance related comments were aimed at alleviating OP’s fear and shifting OP’s perspective towards a better future, such as, “You are in for a long, hard fight but it won’t last forever” or, “Yes you are walking towards the light.” Contrastingly, commenters also expressed concern for OP’s wellbeing and expressed this concern through replies urging OP to, “be safe and don’t hurt yourself or worse.” They expressed concern that the abuser would further hurt the OP. Some commenters also expressed concern the OP may be further victimized by the criminal justice system.

##### Esteem Support

This subtheme consisted of compliments and validation, both of which were aimed at reassuring the OP of their intrinsic worth and that OP’s actions are correct or justifiable (Jensen, 2001).

Compliment were given to OP for seeking help for their IPV or taking steps to leave the abusive relationship. For example, one commenter expressed, “You’ve done a good job by going to the authorities. I’m proud of you.” Similarly, another commenter replied, “Good for you, mister;] All of this is tough, but you’re not only bettering yourself by moving away, but you’re bettering your wife. Way to hold yourself high and protect your life!” Other commenters complimented OP’s parenting skills or complimented their decision to seek child custody, commenting, “Glad to hear your son is doing fine. You did well to get him out of that environment.”

Responses also validated OP’s IPV victimization experience, thoughts, emotions, and actions. Replies included validating male IPV victimization as “absolutely heinous, and unacceptable,” and being just as harmful and abusive as female IPV. Other commenters validated the feeling that any type of violence is abuse. Commenters also validated OP’s feeling of loneliness and sadness as well as OP’s decision to leave the relationship and have a fresh start, as one commenter replied, “That’s definitely understandable. After the load of crap you went through, I don’t blame you at all for wanting to just put it behind you.” Finally, commenters also validated OP’s decision to seek help from police officers and organizations that offer services to IPV survivors.

##### Social Network Support

Social network support consisted of offering companionship for OP. Commenters expressed willingness to listen to OP’s problems or reminded OP that they have a support systems available, as one commenter remarked, “All my love for you at this hard time and remember you aren’t alone.” Commenters urged OP to “stay connected” to the group and keep them updated about the situation. Responses expressed a willingness to connect on a more private one-on-one basis through personal messages (PM), with one commenter reponsind, “If you need to vent feel free to pm me. Here to listen.”

#### Tangible Aid

Theme consisted of “offers to provide tangible resources, services, or assistance to eliminate, solve, or alleviate the problem” (Jensen, 2001, p. 111). Tangible aid was characterized by offers of loans or willingness to help.

In cases where OP indicated they could not leave the abusive relationship because of financial or material barriers (e.g., transportation costs, moving costs, living costs) commenters offered either cash or loan of materials (e.g., loaning a vehicle to help with moving). For example, one commenter indicated that if the OP needed a train ticket “I’ll gladly buy you a ticket anywhere in the UK.” Similarly, another commenter noted they were “more than willing to chip in” for a van to transport OP’s property. In addition to offering loans commenters also expressed willingness to help OP without specifying the exact nature of the help such as the commenter who indicated, “If you’re anywhere near central Bedfordshire, let me know if I can help.”

#### Negative Response

Four subthemes emerged and highlighted the various facets of negative response: (a) Interruption, (b) Victim-Blaming, (c) Minimizing Disclosure, and (d) Questioning Disclosure.

##### Interruption

Interruption consisted of comments that changed the topic and directed the conversation away from OP’s disclosure. This was done primarily through dismissing male IPV as not being a serious issue, or highlighting the higher prevalence of IPV amongst women. For example, one commenter replied, “Statistics show that women are the overwhelming majority of victims of domestic violence.” Similarly, commenters also suggested male IPV cannot be considered seriously given females are much weaker than males. Rather than focus on OP’s disclosure or the reason for the disclosure, the responses distracted from the topic to argue about who the real “victim” of IPV was.

##### Victim-Blaming

Some responses were critical or blamed the victim for the IPV victimization. In one submission, OP disclosed his ex-boyfriend was currently in an abusive relationship prompting a commenter to reply the ex-boyfriend “deserves the abuse” for breaking up with OP and choosing the current abusive relationship. Another commenter replied that, “The queers follow the hate.” Other responses alluded to OP making questionable decisions or missing warning signs, resulting in OP entering into an abusive relationship. Commenters also blamed the OP’s personality for the abuse, suggesting that OP liked playing the victim card, are a pushover, or has a low self-image which predisposed them to stay in the abusive relationship, one commenter responding, “you need help for your self-esteem problem.” Still others mentioned that OP may have codependency issues or mental health disorders ( e.g. Down syndrome).

##### Minimizing Disclosure

Some commenters minimized the disclosure by either making jokes about the abuse or with sarcastic replies. Referring to the reason OP hasn’t left the abusive relationship, one commenter replied, “I’m going to guess OP’s wife is smoking hot.” Some commenters also made jokes about the abuse, for example, when OP disclosed being physically abused with a broom by his abusive girlfriend, in front of his friends, one commenter replied, “Get a broom, you two,” resulting in a reply chain of other commenters making fun of the abuse using broom related puns. Other commenters provided advice that were meant to be taken as a joke, such as, “maybe you should hurry up with the sandwich?” or “sometimes, the simplest solution [is the best solution](http://snookipunch.com/).”

##### Questioning Disclosure

Commenters questioned OP’s version of the abuse or implied there was more to the story than what the OP had posted. In response to OP’s disclosure of a male friend’s IPV experience, one commenter replied, “I’m guessing your friend isn’t telling the full story and there was more information that the police had access to that you don’t.” Another commenter replied, “just cuz you get the shit beat out of you doesn’t mean you’re the victim, either way.”

#### Self-Defence

The theme of self-defence consisted of discussion regarding OP’s right to defend themself and what constitutes as self-defence. The conversation centered on whether males have the right to retaliate as a form of self-defence. Some commenters, both male and female, believed males have the right to hit back; one female responded, “if I’m fighting with some guy (boyfriend, say) and I hit him. . . I wouldn’t be at all surprised/offended that I got hit back. You’re asking for it when you put your hands on someone else.” Similarly, another commenter indicated:

Most people believe that if a guy is hitting you, you should hit him back in self defence. The same applies to women, many are perfectly capable of causing damage and are old enough to figure out that attacking someone is likely to result in retaliation

Other commenters believed females are physically weaker and therefore male IPV suvivors should not be retaliating with violence and instead attempt to either leave the situation or restrain the female perpetrator as a preventive measure. One commenter expressed, “the first thing he should try is to escape the situation if he can, and secondarily try to restrain her from continuing to hit [him].” Still others believed that even restraining the female abuser would constitute violence and the only course of action would be to leave the situation, “restraining a woman with whom you are in a romantic relationship is considered domestic violence in every jurisdiction in North America.” Accordingly, some commenters expressed they were afraid any form of self-defence would be construed as violence and lead to OP being arrested. Others indicated how socially ingrained notion of “never hit a woman” prevented them from defending themselves: this has been explored further elsewhere (Sivagurunathan et al., 2021). Some commenters posited this socially ingrained attitude resulted in males not knowing how to respond to the abuse by their female partners.

#### Reciprocal Disclosure

Disclosure by OP elicited reciprocal disclosure by some commenters. Reciprocal disclosure included both self-disclosure as well as disclosure of IPV experience of friends and family. When commenters disclosed IPV experiences of friends and family they recounted both having directly witnessed the IPV victimization or injury as well as having heard about the abuse second-hand. Reciprocal disclosures seemed to serve two purposes; reciprocal disclosure was used to either supplement suggestions/advice, or as way to express that their understanding/empathy resulted from previous exposure to IPV. Some reciprocal disclosure only served one purpose or the other, while in other cases they served both purposes. One commenter, using disclosure to supplement their advise OP to seek help from police and charities, replied:

I’ll say the same thing to you that I’d say to any woman in the same situation (my sister got out of an abusive relationship a few years ago and is now happy) - it’s not your job to prove it. Talk to the police (and not for nothing but local council and relevant charities), they’ll do anything they can to help.

Another commenter indicated they understood the experience of the male IPV survivors because they had faced similar situation, replying, “Hey man. This was my life to a TEE 6 years ago. Hang in there. I know it’s hard but trust me. Your life is going to get so much better.”

## Discussion

Given the challenges in studying IPV in males, this study provides a unique glimpse into their disclosure experience. Findings demonstrating some issues mirroring the experiences of female survivors of IPV, while others are unique to males. This study identified a wide range of discourses on male IPV disclosures on Reddit, reflecting a number of ways that online respondents react to disclosure by male IPV survivors, including both positive aspects of social support and negative responses.

Given Reddit has an average active user count of 430 million users a month and is used predominantly by young males (Barthel et al., 2016), the number of submissions related to male IPV disclosure is surprisingly limited. This may reflect both the lower prevalence of IPV with men as victims and the ongoing stigma around male IPV victimization. The quantitative content analysis reinforce previous findings that males are reluctant to identify the IPV as victimization (Brooks et al., 2020; Machado et al., 2016). Notions of hegemonic masculinity can influence males’ narratives around victimization (Corbally, 2015; Durfee, 2011); even in cases where males are victims of violence perpetrated by other males (Burcar & Åkerström, 2009). Subsequently, it is possible males who disclosed on Reddit described their experiences of IPV using terminology that was not framed as male IPV and allowed men to “conform to ideals of hegemonic masculinity” (Brooks, Martin, Broda and Poudrier, 2020, p.12). Therefore, the limited amount of submissions may be due to the limitations of the search strategy which used a narrow set of keywords. The limited number of disclosures may also be attributed to males perceiving Reddit as being less conducive to sensitive disclosures due to its negative culture. Unlike health forums, which are often heavily moderated and aim to foster positivity, Reddit sustains many fringe communities whose leanings tend towards shock value through sexism and racism (Kilgo et al., 2018). The toxic culture exhibited by some of the subReddits may have served as a barrier to males disclosing their IPV experiences on Reddit. Future research should aim to explore males’ IPV disclosure on other SNSs (e.g., Twitter, Facebook) and male IPV support forums to determine if the types of responses vary. Strengths and limitations associated with forums and SNSs, as well as different userbase, may result in unique disclosure processes and responses that did not emerge in the current sample.

The results reinforce previous findings (Hines & Douglas, 2010; Machado et al., 2016; Walker et al., 2020) on the types of abuse experiences by male IPV survivors which included physical, emotional, and verbal abuse, as well as controlling and isolating behavior. These types of IPV experiences are similar to those experienced by female IPV survivors (Ansara & Hindin, 2010), however, rates and specific patterns of IPV may be gendered.

Speaking about the outcome of past disclosures, males indicated they had received both positive and negative response to their disclosure. However, similar to previous studies (Douglas & Hines, 2011; Machado et al., 2016; McCarrick et al., 2016), many of the males mentioned having received unhelpful and negative response from police and the justice system. These unhelpful responses are similar to female IPV survivors’ experiences with the legal systems (Meyer, 2011; Ragusa, 2013). Despite these negative experiences or perceptions, male IPV survivors were advised to seek help from police officers and the judicial system.

Commenters’ replies to the disclosure contained negative responses such as victim-blaming, jokes about male IPV and sarcastic responses, as well as replies questioning the OP’s version of the event. These negative responses may be rooted in the prominent social narrative of male dominance and ideas related to who can be a “victim.” Negative responses may serve to further victimize males who are disclosing their experiences on Reddit and act as barrier to future disclosure attempts. However, the majority of the responses by commenters were supportive, reflecting previous findings which reported male IPV survivors were satisfied with the support they received from informal sources (Machado, Hines and Matos, 2016). Research with female IPV survivors indicate that supportive response to the disclosures of IPV had positive mental health impacts (Edwards et al., 2015). The limited research addressing the effects of positive and negative responses on male IPV survivors show similar findings. For example Douglas and Hines (2011) reported positive help-seeking experiences resulted in lower levels of alcohol abuse while negative experiences were associated with higher rates of post-traumatic stress disorder (PTSD). Therefore, the findings from the current study suggest disclosing IPV online may have beneficial impacts for male IPV survivors.

It is important to consider some of the replies in a broader context. Many of the commenters advised OP to leave the abusive relationship. Being told to leave the abusive relationship may result in positive or negative outcomes depending on the survivor’s desire to leave the relationship. Edwards et al. (2015) reported that being told to leave the abusive relationship was positively correlated with both leaving intention and post-traumatic stress symptoms amongst female IPV survivors. Females are also less likely to seek formal intervention for the abuse if they were told to leave the abusive relationship by informal helpers (Fugate et al., 2005). Therefore, advice to leave the abusive relationship may have conflicting effects on mental health and wellbeing. No studies to date have examined the effect of advice to leave a relationship on male IPV survivors’ mental health or intention to leave, therefore, further studies are needed. Similarly, commenters also advised OP to “man up” and leave the relationship. Commenters’ suggestion to “man up” could be construed as a negative comment meant to further emasculate the male survivors, or as a positive call to arms, to channel the perceived inherent masculine traits such as strength and protectiveness to make positive changes in one’s life. While a majority of studies to date have focused on male gender role stress or toxic masculinity and the impact of masculine ideologies on problems in various facets on male health and wellbeing, a growing number of researchers have called for the need to examine masculinity using the positive psychology positive masculinity paradigm (McDermott et al., 2019). It may be argued that calls to “man up” by commenters in the current study are meant to evoke “societal expectations that men [. . .] stay calm in the face of adversity, display courage, power through obstacles” (McDermott et al., 2019, p. 14).

While some forms of stigma may be common to both male and female IPV survivors, other forms of stigma are unique to male IPV survivors. One form of stigma unique to male IPV survivors seems to be related to discourse around self-defence. Studies on self-defence motives for IPV perpetration indicate that both males and females report using physical violence as a form of self-defence (Leisring & Grigorian, 2016). However, social stigma and beliefs such as “you don’t hit girls” may limit male IPV survivors utilizing physical self-defence startegies. There has been extensive research on female IPV survivors’ self defence strategies (Downs et al., 2007; Jordan & Mossman, 2019). However, research on effective self-defence strategies for male IPV survivors are limited. Further research and education on effective self-defence strategies, including nonphysical self-defense strategies, for male IPV survivors are warranted.

One of the prevalent themes that emerged in both the disclosure and response was fear of false accusation. Both OP and commenters mentioned being falsely accused of IPV perpetration (as a form of legal and administrative violence) or being threatened of false allegation (possibly as a tactic to threaten and silence the male survivors), reinforcing previous research on male IPV survivors (Corbally, 2015; Morgan & Wells, 2016; Walker et al., 2020). While research on false IPV allegations is limited, the findings are equivocal with researchers finding both male and females may use false allegations to control their partners (Mazeh & Widrig, 2016). In the current study commenters advised using tape recorders and security cameras to record the abuse, as tactic to prevent false allegations and as a way to establish victimhood. These findings may point to men’s fear of invalidation, a phenomenon whereby “disclosure of abuse was blatantly ignored or discounted” (Lutenbacher et al. 2003, p. 60).

### Strengths and Limitations

The study is not without its limitations. Submissions included in the study were gathered using three specific search terms related to male IPV and as such may not have captured the entire spectrum of conversation on male IPV. Including general search terms may result in additional data. Furthermore, given the relative anonymity of the platform we could not make any assumptions about the demographics of the OPs or the commenters. Subsequently, the anonymity of the Reddit posts also precluded our ability to interrogate the data further for any meaningful effects of sexual orientation, age, or other demographic variables. This is a reasonable area for future research. Finally, due to the nature of data collection, we were unable to explore reasons for commenter’s posts or ask for further clarifications, as such there is a potential to misinterpret some statements out of context.

## Conclusions

Anonymity provided by SNSs may allow men to disclose their IPV experiences without the risks associated with in-person disclosures. The study contributes to further understanding of male IPV disclosure and responses within the context of a SNS. Findings identified online disclosure of male IPV experiences result in variety of positive forms of social supports and some negative responses. Negative responses that ridicule or dismiss the disclosure may be related to masculine role expectations.

## Notes

1 According to redditinc.com, accessed January 26, 2020.
